# Rethinking learning from incidents in healthcare: a Safety-II model integrating work-as-imagined, work-as-done, and work-as-experienced

**DOI:** 10.3389/frhs.2026.1884750

**Published:** 2026-07-27

**Authors:** Nawal Khattabi, Axel Ros, Matthew Carey, Aled Jones

**Affiliations:** 1 Primary Health Care Corporation, Doha, Qatar; 2 Faculty of Health, Plymouth University, United Kingdom; 3 Jönköping Academy for Improvement of Health and Welfare, School of Health and Welfare, Jönköping University, Jönköping, Sweden

**Keywords:** healthcare systems, incident investigation, learning from incidents, patient experience, patient safety, resilient healthcare, Safety-II, work-as-done

## Abstract

Healthcare organizations increasingly investigate patient safety incidents using systems thinking. However, even investigations that claim to adopt a systems approach frequently revert to linear, event-focused reasoning concluding on deviations and recommending further training. A foundational question remains unresolved: when an incident review identifies a “deviation,” does this represent exceptional behavior or the system's typical mode of operation that generally succeeds under varying conditions but occasionally fails? Without this distinction, explanations for incidents tend to focus on individual non-compliance rather than understanding the everyday adaptations that enable care delivery in a complex adaptive system. Consequently, incident reviews that promote system learning often continue to recommend retraining, reminders, and reinforced compliance. In this Perspective, we propose a Safety-II model for incident review structured around three analytical lenses: work-as-imagined, work-as-done, and work-as-experienced—the last capturing what only patients and families can witness, longitudinally, across settings and professional boundaries. We introduce a prevalence-indexed analysis of work-as-done, in which longitudinal electronic medical record and operational data are used to establish whether a flagged practice represents exception or norm, while remaining transparent about the gap between documented and enacted work. We position this contribution alongside recent advances in incident analysis and patient partnership, and set out concrete proposals for regulators, healthcare organizations, and researchers.

## Introduction

The principle of avoiding harm is foundational to medicine, traceable at least to the Hippocratic principle “to help or at least do no harm” (1). Centuries later, Florence Nightingale extended this individual ethical commitment into an organizational concern, observing: “It may seem a strange principle to enunciate as the very first requirement in a hospital that it should do the sick no harm” (2). Published in 2000, “To err is Human,” a landmark document that influenced healthcare policy, acknowledged the inherently complex nature of healthcare and, among other recommendations, emphasized the importance of learning from patient safety events and implementing system wide improvements (3). Despite more than three decades of increased attention, policy development, and investment in patient safety, healthcare systems have achieved limited and uneven reductions in patient harm (4, 5). One explanation for the relatively slow progress in advancing patient safety is that multiple issues have been identified in the process of learning from safety incidents. These include a lack of depth in incident review, absence of evaluation and validation of the techniques used in reviewing the incidents (6–8), as well as a failure to systematically compare work-as-imagined with work-as-done even in investigations claiming a systemic approach (9, 10). Further issues include hindsight bias (11, 12), a narrow focus on compliance, and the comparison of practice during the event, which unfolds in a dynamic and multi-factorial context, with guidelines and policies that are static in time (13–16). As a result, many investigations become procedurally complete yet epistemically limited, identifying deviations without establishing whether these reflect abnormal behavior or the system's normal operating mode. This limits learning by explaining harm through non-compliance rather than by understanding how adaptive practices usually succeed and occasionally fail in complex systems (16, 17). Over time, patient safety thinking has evolved from a traditional approach which understood incidents as arising from linear chains of error and identifiable root causes, to a more holistic, dynamic systems view, recognizing healthcare as a complex adaptive system. Contemporary approaches emphasize how outcomes emerge from interactions between people, processes, technologies, and context, focusing not only on failure but also on everyday adaptations and resilience that enable care to succeed under varying conditions (9, 13, 18).

### Systems-aware in name, linear in practice

Wiig, Braithwaite, and Clay-Williams (19) urge the healthcare sector to integrate safety science theories and frameworks into current limited safety investigation methodologies to address some of these issues. These issues reflect assumptions associated with the dominant Safety-I approach. Safety-I defines safety primarily as the absence of adverse outcomes and tends to investigate incidents by tracing the event backwards to identify failures, deviations, or causes to be corrected (20). Safety-II starts from a different premise: in complex healthcare systems, safety depends on the capacity of people, teams, technologies, and organizations to adjust performance under variable and often imperfect conditions (17, 21). From this perspective, the same performance variability that enables care to succeed in everyday practice may, under different conditions, contribute to harm. Incident review should therefore examine both the index event and the ordinary functioning of the same process, asking how care usually succeeds as well as how it failed on a given occasion (9, 17, 22). This challenges traditional incident review methods that assess harm primarily in the isolated context of the event. Policy makers, process designers, and administrators cannot anticipate all circumstances or fully account for the dynamic nature of health care delivery (21). Practitioner may therefore adjust a procedure to meet patients’ needs in circumstances not predicted or supported by formal guidance. The review question then becomes not only whether practice departed from the policy or guideline, but whether the policy, resources, workflow, and operational conditions made safe practice achievable.

Two recent contributions converge with these concerns without fully resolving them. Bowditch et al. (10) demonstrate in an empirical analysis of Australian incident investigations that even when investigators describe their approach as systems-based, the analytical patterns of contributing factors and recommendations remain predominantly linear and event-bound. Vincent et al. (23), in the new edition of the London Protocol, extend the analytical horizon, foreground patient and family partnership, and call for the examination of good care alongside adverse events. Both contributions advance the field. Neither, however, addresses a prior question: when an investigator identifies a deviation from policy, how can they determine whether it represents an exceptional departure or the system's normal operating mode? Without this determination, incident reviews explain harm through individual non-compliance or technical failures and miss identifying the variability that ordinarily makes care succeed in complex adaptive system.

### Three lenses in incident reviews: work-as-imagined, work-as-done, work-as-experienced

We propose that a Safety II-based incident review model would better capture and account for such deviation and unpredictability present across the various types of healthcare work. This model captures work-as-done (the actual practices and actions of individuals and processes), work-as-imagined (the policies, guidelines and planned procedures), and work-as-experienced (the communication and care received by the patient) to understand different aspects and perspectives of incidents occurring with a complex system. The concepts work-as-done and work-as-imagined in incidents review is influenced by the work of Hollnagel (20) and Braithwaite, Wears, and Hollnagel (13), who pioneered the fundamental principles of Safety-II, Resilient Healthcare and the crucial need for understanding and reconciling the gap between the work-as-imagined and work-as-done. Work-as-experienced is added in this model to capture patient experience as an analytical distinct source of system information. Patients and families are often the only actors who experience care longitudinally across setting, services and professional boundaries; they observe coordination, communication, and continuity from a vantage point that staff narratives and clinical records cannot replicate (16, 23, 24). The value of work-as-experienced is therefore not only ethical or relational; it is epistemic, because patients and families can reveal aspects of safety that may remain invisible in formal records and professional accounts. Recent co-designed approaches to involving patients and families in incident investigation demonstrate that these accounts can surface contributory factors that formal detection methods miss, that how investigations elicit and credit them shapes what is learned (25, 26). This indicates that work-as-experienced should be an indispensable analytical lens for understanding how adaptive interactions, coordination gaps, and cumulative risk emerge over time, insights that policies and professional accounts alone cannot capture. In this way the three lenses bring together the perspectives of those who plan care, those who deliver it, and those who receive it, instead of privileging any single account. Table 1 summarizes the analytical contribution of Safety-I, Safety-II, and Work-as-Experienced in incident reviews.

**Table 1:** Comparative analysis of Safety-I, Safety-II, and work-as-experienced analytical dimensions. The Safety-I and Safety-II dimensions build on Hollnagel's contrast of the two Safety paradigms (20, 22) and the resilient healthcare literature (9, 17, 21), with the dimensions elaborated for incident review. The work-as-experienced dimension extends this comparison to the patient and family perspective (30).

Dimension	Safety-I approach	Safety-II approach	Work-as-experienced lens
Definition of Safety	Safety as the near-absence of adverse outcomes; the goal is to minimize the frequency of things going wrong	Safety as the capacity to sustain successful performance across varying conditions, so that intended outcomes obtain as often as possible	Care feels safe, coordinated, and respectful to patient and family
View of Human Performance	Humans as liability; source of error	Humans as resource; source of system resilience	Patients as co-producers who adapt and compensate for system gaps
Focus of Analysis	Failures, errors, deviations from protocol	Performance variability: what enables success and failure	Care journey coherence and coordination; communication; resources, feeling heard and respected
Primary Data Source	Incident reports; clinical records; staff interviews	Similar incident reports, performance indicators on relevant processes, clinical record beyond adverse event timeline, direct observation, ethnography, staff narratives of everyday work and the adverse event	Patient and family narratives; experience data; complaint patterns
Explanatory Model	Linear causality; root cause; chain of errors	Functional resonance; emergence; systemic interactions	Narrative coherence; emotional impact; relational quality
Orientation	Reactive; retrospective	Breadth and depth; Proactive and reactive;	Longitudinal; attentive to care continuity over time
Response to Variation	Variation is deviation; should be reduced	Variation is adaptive; should be understood to inform policy and system enhancement	Variation in patient experience signals system performance, early warning signs of potential incidents; should be explored
Typical Recommendations	Retrain staff; add warnings; strengthen compliance	Redesign systems; support adaptive capacity; align work-as-imagined with work-as-done	Improve communication; patient engagement, care coordination, safe transitions, emotional needs;
Investigative Questions	What went wrong? Who is responsible?	How did this outcome emerge? What enabled/constrained adaptation?	How did patient experience care? Where did they feel gaps?
Learning Orientation	Learn from failure to prevent recurrence	Learn from success and failure to strengthen resilience	Learn from patient insights to understand care coordination and transition, humanize care and acknowledge the co-creation of health outcomes
Patient Role	Subject of investigation; source of harm data	Component of sociotechnical system	Expert on own experience; partner in learning; witness across transitions
Quality of Evidence	Documentary; verifiable; objective	Objective (System Data, Performance data over time). Contextual; interpretive; acknowledges complexity	Experiential; subjective but valid; captures what records miss
Outcome Metric	Incident rates; harm counts; compliance percentages	Adaptive capacity; system resilience indicators	Patients experience scores; trust restoration; litigation rates

**Figure 1:**
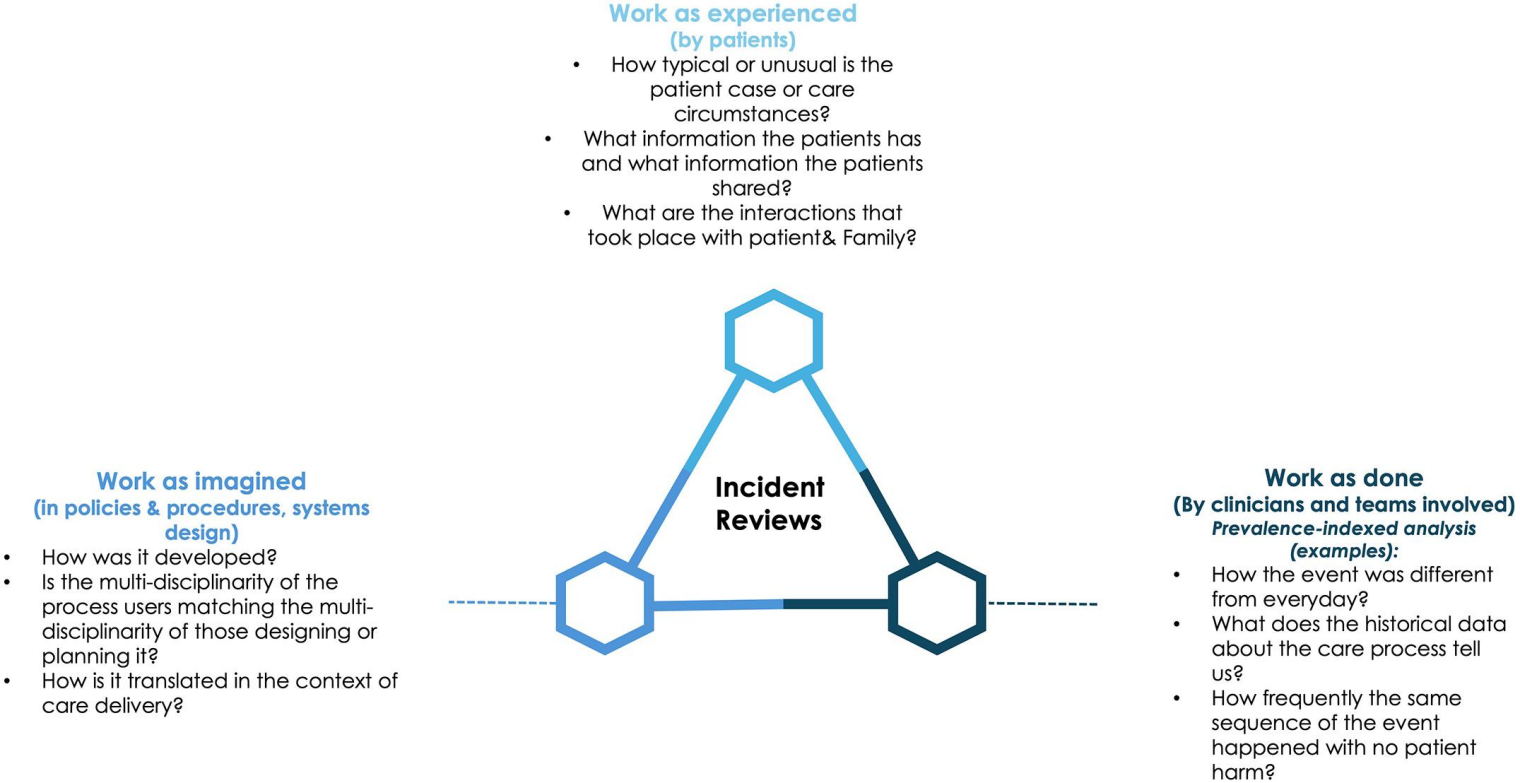
Safety II-inspired incident review model drawing upon the principles proposed by Hollnagel (20) and Braithwaite, Wears, and Hollnagel (13, 21).

Figure 1 presents the proposed model, organized around the three lenses and the type of questions each brings to an incident review. Building on these distinctions, this model extends beyond expanding data sources, time horizons, or perspectives (23). It introduces a prevalence-indexed analysis of work-as-done; this can be achieved using longitudinal electronic medical record trends and other technologies and data sources to determine whether practices identified as “deviations” in adverse events represent a deviation or normalized system functioning. Without establishing how frequently such practices occur over time and across settings, incident investigations risk misclassifying routine adaptive drift as individual error, thereby perpetuating Safety-I logic under a Safety-II label.

### Prevalence-indexed analysis of work-as-done

Understanding the way work is: 1) informed by policies, 2) executed by clinicians whether during the incident or in normal conditions, and 3) experienced by the patients, would help identify discrepancies leading to insights for improving safety, efficiency, and organizational learning. The analytical contribution of this model lies not merely in gathering data from these three lenses but in reconciling them to determine whether practices identified during investigation represent routine system adaptation or true deviation. See Box 1 for an example where this is illustrated in a case of medication error.

BOX 1 **Prevalence-indexed analysis of a medication error: from individual deviation to system pattern (*The figures below are illustrative and do not represent empirical thresholds*).**

**Table:** 

De Micco et al. (27) illustrate how a surface-level evaluation might conclude that the nurse simply “was not paying attention,” while the systemic approach considers more: Was the drug label hard to decipher? Was the electronic prescribing system poorly designed to yield confusing warnings? Was the nurse working an inordinately extended shift exhausted? Were routine double-checking protocols missing or used sporadically? This systemic inquiry may identify potential contributory factors but remains event-bound and doesn't determine whether these represent routine system functioning or aberrant conditions. Through the Safety-II lenses of this model, those questions would be framed as prevalence-indexed analysis: • How often do nurses on this unit work extended shifts exceeding safe thresholds? If 73% of shifts in the past quarter exceeded recommended hours, the fatigue is not an individual circumstance but a system-level condition, which redirects the review from the individual toward staffing and scheduling arrangements. • How frequently are double-checking protocols bypassed across this unit, this hospital, this health system? If electronic medical record data show that 40% of high-risk medication administrations proceed without documented second verification, the omission is not an isolated lapse by one practitioner but a normalized practice, which raises more questions: is the second verification protocol unworkable as written, is it adequately resourced or trained for, or can it be safely redesigned. • How many similar near-misses involving this drug label or prescribing system interface have occurred without harm? If trend data reveal 200 near-miss events over 18 months involving the same confusing alert, the harm event is not explained by the alert design alone—the question becomes why the system's compensatory adaptations (which succeeded 200 times) failed this time.

Without quantifying the prevalence of work-as-done across time and settings, investigators cannot distinguish whether flagged practices represent single individual behavior or the system's usual operating mode (9, 15). The same variability labelled “error” after harm may be the very adaptation that enables success under conditions the system routinely generates. This is the analytical step that transforms Safety-II from philosophy into investigative method: not merely acknowledging the gap between work-as-imagined and work-as-done, but establishing whether a practice represents exceptional deviation or normalized drift (28). This distinction is also what could justify a proportionate change, as a single incident could be dismissed as an exception, whereas a recurring pattern would provide the basis for revising a guideline or redesigning a process instead of defaulting to retraining.

Two methodological cautions warrant explicit acknowledgement. First, electronic medical record data and operational performance indicators reflect documented practice and not enacted practice, and are themselves shaped by documentation conventions, copy-forward behaviors, structured-field constraints, and selective recording (29). What appears in the record is therefore an artefact of work-as-done filtered through institutional documentation logic, and not work-as-done itself. Second, prevalence quantified from these data should be triangulated with direct observation, staff narratives, and patient-reported information and not treated as ground truth. The intent is not to replace qualitative inquiry with frequency counts, but to anchor investigators’ judgements about what is normal and what is exceptional in evidence wider than the index event. Surfacing work-as-done in this way depends on an environment where honest accounts of everyday practice can be shared and examined for learning.

## Discussion: from aspiration to a review method

Advancing learning from incidents requires more than extending investigative timelines or incorporating additional perspectives. It requires theory-explicit approaches capable of distinguishing exceptional deviation from normalized adaptive drift, and of judging whether investigations generate learning that is explanatory, system-oriented, and actionable in complex adaptive healthcare systems. At present, healthcare lacks an integrated Safety-II framework and critically, lacks agreed criteria for assessing the quality of incident investigations and the learning they produce.

Addressing healthcare's perennial “no time” constraint demands a shift from episodic, event-bounded reconstruction toward evidence-informed characterization of work-as-done across time and settings. Electronic medical records, longitudinal performance indicators, and aggregated incident data constitute underutilized system resources for establishing whether practices highlighted during investigations reflect normalized system drift or isolated deviation. Crucially, this does not impose additional burden on clinical or corporate staff for data collection: the data already exist within routine documentation and reporting infrastructures. What is currently missing is not data, but analytical and evaluative capability. Incident investigations require structured ways to interrogate routine operational data, to triangulate work-as-imagined, work-as-done, and work-as-experienced, and to make explicit judgements about the depth, coherence, and system relevance of the learning generated.

We therefore position this Perspective as a methodological bridge between Safety-II theory and operational incident review practice. To advance incident reviews to meaningful source of learning, we recommend three concrete developments. First, healthcare regulators and patient safety bodies should establish minimum quality criteria for the conduct of incident investigations that explicitly require triangulation of work-as-imagined, work-as-done, and work-as-experienced, and that distinguish prevalence-indexed analysis from event-bound reconstruction. Second, healthcare organizations and informatics teams should support with analytical tooling, including, where appropriate, machine learning approaches that can characterize work-as-done from longitudinal record data while remaining transparent about documentation biases, enabling prevalence-indexed analysis at scale. Third, patient and family voices should be repositioned in incident investigation governance, not as supplementary input but as a distinct analytical lens, with methods, training, and reporting structures commensurate with that role (30).

## Data Availability

The original contributions presented in the study are included in the article/Supplementary Material, further inquiries can be directed to the corresponding author.
